# Hypoxia induces phenotypic and metabolic shifts in endophytic *Flavobacterium* sp. 98

**DOI:** 10.1093/ismejo/wraf269

**Published:** 2025-12-06

**Authors:** Xinya Pan, Janne J Hageman, Daan A Weits, Lhais Caldas, Somayah S Elsayed, Lina M Bayona, Gilles P van Wezel, Roeland L Berendsen, Víctor J Carrión, Jos M Raaijmakers

**Affiliations:** Department of Microbial Ecology, Netherlands Institute of Ecology (NIOO-KNAW), 6708 PB Wageningen, The Netherlands; Institute of Biology, Leiden University, 2333 BE Leiden, The Netherlands; Experimental and Computational Plant Development, Institute of Environment Biology, Utrecht University, 3584 CH Utrecht, The Netherlands; Experimental and Computational Plant Development, Institute of Environment Biology, Utrecht University, 3584 CH Utrecht, The Netherlands; Department of Microbial Ecology, Netherlands Institute of Ecology (NIOO-KNAW), 6708 PB Wageningen, The Netherlands; Institute of Biology, Leiden University, 2333 BE Leiden, The Netherlands; Institute of Biology, Leiden University, 2333 BE Leiden, The Netherlands; Department of Microbial Ecology, Netherlands Institute of Ecology (NIOO-KNAW), 6708 PB Wageningen, The Netherlands; Institute of Biology, Leiden University, 2333 BE Leiden, The Netherlands; Plant–Microbe Interactions, Institute of Environmental Biology, Department of Biology, Science4Life, Utrecht University, 3584 CH Utrecht, The Netherlands; Department of Microbial Ecology, Netherlands Institute of Ecology (NIOO-KNAW), 6708 PB Wageningen, The Netherlands; Institute of Biology, Leiden University, 2333 BE Leiden, The Netherlands; Departamento de Microbiología, Facultad de Ciencias, Universidad de Málaga, 29010 Málaga, Spain; Departamento de Protección de Cultivos, Instituto de Hortofruticultura Subtropical y Mediterránea “La Mayora”, IHSM-UMA-CSIC, 29010 Málaga, Spain; Department of Microbial Ecology, Netherlands Institute of Ecology (NIOO-KNAW), 6708 PB Wageningen, The Netherlands; Institute of Biology, Leiden University, 2333 BE Leiden, The Netherlands

**Keywords:** plant-endophyte interactions, hypoxia, untargeted metabolomics, root oxygen measurement, *Flavobacterium*

## Abstract

Oxygen plays a crucial role in shaping microbial physiology, functions, and behavior. Endophytic bacteria, residing within plant tissues, inhabit microenvironments where oxygen availability can be limited. However, the magnitude of hypoxic conditions in the endosphere and how these affect functional microbial traits are largely unknown. Here, we showed with a microsensor that oxygen levels in roots of sugar beet seedlings drop drastically to variable, low oxygen levels when going from epidermal to endodermal root tissue into the vasculature. Subsequently, we investigated phenotypic and metabolic responses of endophytic *Flavobacterium* sp. 98 at oxygen levels of 100 ppm. Under these low oxygen conditions, *Flavobacterium* sp. 98 showed reduced growth, enhanced motility, and an altered extracellular metabolite profile. *Flavobacterium* sp. 98 colonies spread out in response to oxygen limitation and more effectively restricted hyphal growth of the sugar beet root pathogen *Rhizoctonia solani* than *Flavobacterium* sp. 98 grown at ambient oxygen conditions. Exometabolome analysis revealed enhanced accumulation of lysophosphatidylethanolamine (lysoPE) and *N*-acetyl-phenylalanine under low-oxygen conditions, along with a reduced level of the antifungal compound 5,6-dimethylbenzimidazole. These responses reflect physiological and metabolic plasticity of *Flavobacterium* sp. 98, highlighting significant changes in the expression of specific traits under hypoxic conditions. Our findings provide insights into niche-adaptive strategies of endophytic bacteria and pinpoint functional traits in microbe-plant interactions operating inside plant tissue.

Oxygen makes up ~21% of the modern Earth’s atmosphere, a level often taken for granted and typically assumed in standard laboratory settings. However, dynamic oxygen concentrations create environmental micro-niches for microorganisms [1–3]. This was exemplified for the growth and survival of marine *Streptomyces*, where hypoxia triggered shifts in secondary metabolite production linked to extracellular electron shuttling [4]. For plant-associated microorganisms, hypoxia may affect cellular metabolism and signaling [5–7], and shape the interactions with their host plants**.** The root endosphere (internal root tissues) is typically low in oxygen [8], but the impact of oxygen limitation on metabolism of plant-beneficial endophytes is still unexplored**.** Here, we studied the effects of hypoxic conditions on phenotypic and metabolic traits of endophytic *Flavobacterium* sp. 98, a strain that was previously isolated from the endopshere of sugar beet roots and that provided protection against the fungal root pathogen *Rhizoctonia solani* [9]. We hypothesized that *Flavobacterium* sp. 98 may encounter low oxygen concentrations within root tissues and exhibit activities different from those observed under standard laboratory conditions at atmospheric oxygen levels.

We first measured oxygen levels in sugar beet roots with a highly sensitive microsensor and found a substantial drop in oxygen concentrations going from the rhizosphere to the root vasculature of several seedlings (Fig. 1A and B, Supplementary Fig. S7). This indicates the presence of hypoxic micro-niches within sugar beet root tissues that may affect the phenotype and metabolome of *Flavobacterium* sp. 98. Subsequent bioassays were conducted in a closed chamber with oxygen levels set at 100 ppm. Although this oxygen concentration was lower than those measured in the roots, it was selected to maximize the observable responses while still representing a biologically relevant niche in localized and diffusion-limited regions in the root vasculature. We found that hypoxic conditions reduced the growth of *Flavobacterium* sp. 98, which could be restored, in part, upon re-exposure to atmospheric oxygen conditions (Fig. 1C and D). Moreover, the hypoxic conditions triggered enhanced motility of *Flavobacterium* sp. 98 (Fig. 1E and G). As a result of outward colony growth on agar plates at hypoxic conditions, *Flavobacterium* sp. 98 restricted hyphal growth of *R. solani* more than under ambient oxygen conditions (Fig. 1F and H). Hence, under hypoxic conditions that occur inside sugar beet root tissues, *Flavobacterium* sp. 98 appears to compete more effectively with the fungal pathogen.

**Figure 1:**
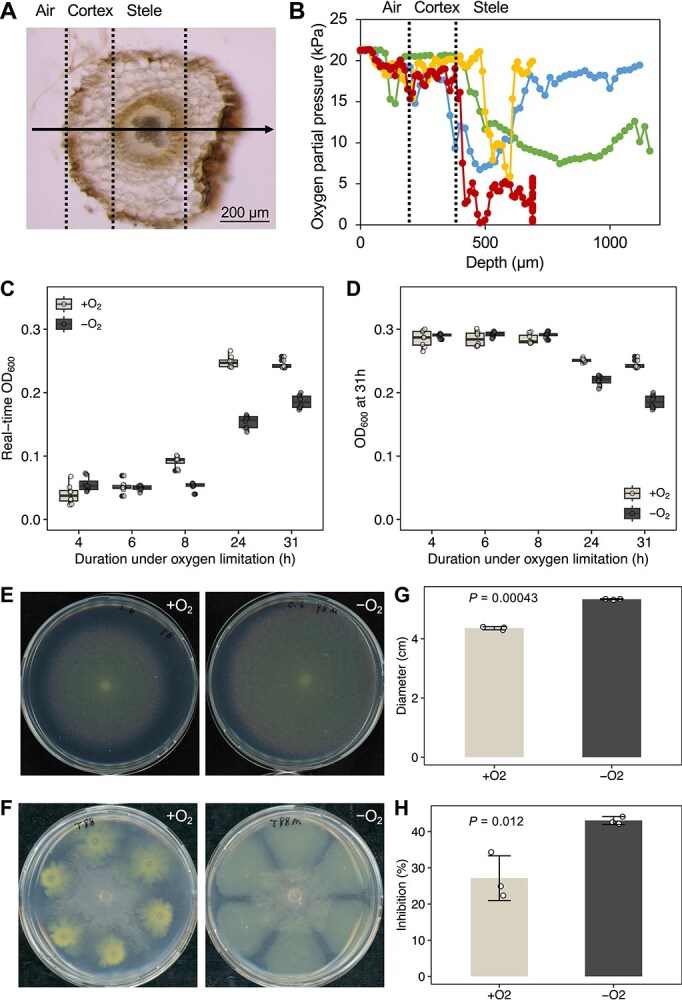
Oxygen gradients in sugar beet root tissue and phenotypic changes of endophytic *Flavobacterium* sp. 98 grown under hypoxic conditions. (A) Representative cross-section of a 2-week-old sugar beet root. The solid line with arrow shows the trajectory of the oxygen microsensor. (B) Combined oxygen profiles from four independent experiments. Oxygen content was measured with a highly sensitive oxygen microsensor at the root-shoot junction of 2-week-old sugar beet seedlings, ~1 cm below the stem base (the site where the fungal root pathogen *R. solani* typically infects). Dashed lines indicate the approximate boundaries from air to cortex to stele as the microsensor penetrated the root tissue. The x-axis represents the depth into the root. (C) Optical density (OD_600_) of *Flavobacterium* sp. 98 cultures grown under atmospheric oxygen (+O_2_) and hypoxic (−O_2_) conditions. The x-axis indicates incubation time in hours. (D) Optical density (OD_600_) of *Flavobacterium* sp. 98 cultures at 31 h after initial inoculation. Ten 96-well plates were inoculated simultaneously and five of them were placed in an oxygen-depleted chamber. To generate different durations of oxygen limitation, the plates were removed from the chamber after 4, 6, 8, 24, and 31 h of incubation at oxygen-limited conditions, respectively. Thereafter, the plates were incubated under atmospheric conditions until the final measurement at 31 h post-inoculation. Control plates were kept under atmospheric conditions for the entire 31 h (+O₂). The x-axis indicates the duration of hypoxic conditions (−O₂). (E, F) Representative images showing colony spreading and antagonistic activity against *R. solani* under normal (+O_2_) and oxygen-limited (−O_2_) conditions. (G, H) Bar plots showing significantly increased gliding motility of *Flavobacterium* sp. 98 and its enhanced inhibition of *R. solani* growth under hypoxic conditions (Student’s t test, *P* < .05). The y-axis represents the diameter of colony spreading (G), or the percentage of hyphal growth inhibition relative to the control without *Flavobacterium* sp. 98 (H). Bars represent mean values ± SD (*N* = 3).

To elucidate the effects of oxygen limitation on bacterial metabolism, we investigated the exometabolome of *Flavobacterium* sp. 98 grown inside or outside the oxygen-depleted chamber (Supplementary Table S1). Untargeted metabolomics revealed 254 differentially abundant mass features between the two oxygen conditions (Fig. 2B; foldchange >2, false discovery rate (FDR)-adjusted *P* < .05). A total of 111 mass features were significantly enhanced, and 143 were reduced in abundance in extracts of *Flavobacterium* sp. 98 grown under hypoxic conditions (Fig. 2C, Supplementary Table S2). Only 108 mass features could be annotated with the CANOPUS module of SIRIUS, suggesting that a large portion of the mass features remain unknown or underrepresented in current databases (Supplementary Tables S2 and S3, Fig. 2D; [10]). Among the “down-regulated” mass features, the antifungal compound 5,6-dimethylbenzimidazole (DMB, 147.0915 mz/3.51 min) was not detected in extracts of *Flavobacterium* sp. 98 grown under hypoxic conditions (Supplementary Fig. S1A). This finding aligns with previous reports showing that DMB synthase activity is oxygen-dependent [11], implying increased DMB production by *Flavobacterium* sp. 98 in oxygenated microenvironments such as root wounds formed during pathogen invasion [12]. Other compounds reduced in *Flavobacterium* sp. 98 extracts at hypoxic levels include putative cytokinins, which may be involved in modulating plant hormone crosstalk (Supplementary Fig. S1B and C; [13]).

**Figure 2:**
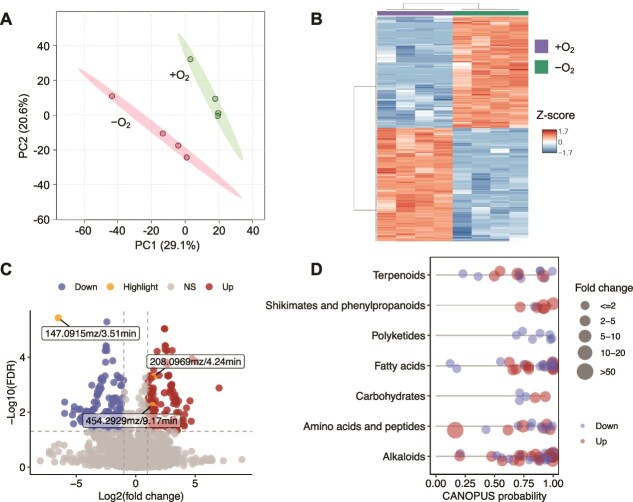
Metabolic responses of endophytic *Flavobacterium* sp. 98 to oxygen limitation. To investigate the exometabolome of *Flavobacterium* sp. 98 grown under hypoxic conditions, we performed comparative metabolomic analysis of extracts from *Flavobacterium* sp. 98 cultures grown on 1/10th strength tryptone soy agar inside or outside the oxygen-depleted chamber. (A) Principal component analysis plot showing that the extracellular metabolite profile of *Flavobacterium* sp. 98 grown under oxygen limitation was significantly altered from that of *Flavobacterium* sp. 98 grown under ambient oxygen levels. Permutational multivariate analysis of variance (PERMANOVA): *R*^2^ = 0.49, *P* = .028; Permutational multivariate analysis of dispersion (PERMDISP): *F* = 0.077, *P* = .776. The grouping variable (oxygen condition) explained 49.7% of the variance of the metabolomic composition of the *Flavobacterium* sp. 98 extracts, with a total of 5305 mass features detected. (B) Heatmap showing the 254 mass features that differed significantly in relative abundance in extracts of *Flavobacterium* sp. 98 grown under hypoxic conditions (foldchange >2, FDR-adjusted *P* < .05). Each row is a significantly differentially abundant mass feature, and each column is a replicate sample (*N* = 4). (C) Volcano plot showing differentially abundant mass features of *Flavobacterium* sp. 98 grown under ambient and hypoxic conditions. “Up” and “down” refer to significantly enhanced (*N* = 111) and reduced (*N* = 143) mass features of *Flavobacterium* sp. 98 under hypoxic conditions (fold change > 2, FDR adjusted *P* < .05). 5,6-dimethylbenzimidazole (147.0915 mz/3.51 min), *N*-acetyl-phenylalanine (208.0969 mz/4.24 min), and 1-16:0-lysoPE (454.2929 mz/9.17 min) are highlighted. (D) Dot plot showing SIRIUS annotations of 108 differential mass features. Each dot is a differential mass feature. Each line represents a compound annotated to a specific chemical class (natural product pathway, y-axis). The x-axis indicates the CANOPUS annotation probability for each mass feature. Dot size reflects fold change, whereas “up” and “down” represent features with increased and decreased abundance under oxygen limitation, respectively.

We focused on the mass features detected at significantly higher abundance under hypoxic conditions, as they may be linked to novel activities of *Flavobacterium* sp. 98 in the plant vasculature. In particular, phenylalanine and phosphoethanolamine (PE) derivatives were found to be significantly enhanced under hypoxic conditions (Supplementary Figs. S2 and S3). Tandem mass spectrometry (MS/MS) analyses including pure standards confirmed the identity of *N*-acetyl-phenylalanine (either l- or d- form, 208.0969 mz/4.24 min) and 1-16:0-lysoPE (454.2929 mz/9.17 min) observed in *Flavobacterium* sp. 98 extracts (Supplementary Fig. S4). Phenylalanine is a precursor of various metabolites contributing to plant growth, development, and defense [14, 15]. Recent studies have elucidated the complete biosynthetic pathway of phenylalanine-dependent salicylic acid (SA) in plants [16–18]. Our findings suggest that the SA pathway could be modulated by endophytic bacteria such as *Flavobacterium* sp. 98 in oxygen-limited roots. Moreover, its derivative *N*-acetyl-l-phenylalanine has been reported to accumulate in walnut roots after inoculation with arbuscular mycorrhizal fungi upon drought stress [19]. Similarly, lysophospholipid derivatives such as 16:0-lysoPE, enhanced in the *Flavobacterium* sp. 98 exometabolome under hypoxic conditions, have also been shown to promote plant growth and health [20, 21]. These reports suggest that *Flavobacterium* sp. 98 metabolites increased under hypoxic conditions could potentially contribute to the regulation of plant growth and/or defense, but their accumulation and relevance *in planta* will require future validation. Subsequent assays revealed that *N*-acetyl-d- or l-phenylalanine or 1-16:0-lysoPE did not inhibit *R. solani* hyphal growth *in vitro*, further indicating that their protective effects could be indirect through reprogramming plant metabolism and priming plant defense [19, 21].

For the intestinal pathogen *Campylobacter*, lysophospholipids were shown to be essential for motility under low-oxygen conditions [22]. We hypothesized that hypoxia-induced metabolites might similarly contribute to *Flavobacterium* sp. 98’s motility. Results of our bioassays, however, provide no evidence for increased motility of *Flavobacterium* sp. 98 in the presence of *N*-acetyl-d- or l-phenylalanine or 1-16:0-lysoPE under atmospheric oxygen conditions (Supplementary Fig. S5A). In addition, *Flavobacterium* sp. 98 growth was enhanced by these compounds at ambient oxygen levels (Supplementary Fig. S5B), suggesting that their accumulation upon hypoxia may serve as an alternative nutrient source. Genome mining revealed that *Flavobacterium* sp. 98 harbors a *pldA* homologous gene encoding phospholipase A, which is essential for lysophospholipid biosynthesis [22]. Moreover, pathway reconstruction indicated that *Flavobacterium* sp. 98 has the complete capacity for phenylalanine biosynthesis via the shikimate pathway (Supplementary Fig. S6), in addition to several putative *N*-acetyltransferases. These genes are promising targets for mechanistic investigation and validation of the roles of their encoded metabolites in *Flavobacterium* sp. 98’s functioning in and adaptation to hypoxic niches.

In conclusion, this exploration of the oxygen-limited environment inside plant roots provides new insights into the phenotypic and metabolic adaptations of endophytic *Flavobacterium* sp. 98. Enhanced motility under low-oxygen conditions may facilitate endophytic colonization of *Flavobacterium* sp. 98 and contribute to niche exclusion of fungal pathogens [23]. We also reported the effects of hypoxia on the *Flavobacterium* sp. 98 exometabolome, highlighting changes of several metabolites with putative functions in plant growth and defense. Overall, this work advances our understanding on how overlooked environmental and internal cues such as oxygen availability may shape plant-endophyte interactions.

## Supplementary Material

SuppData_hypoxia_isme_2025_XP

## Data Availability

The data from this article are available in the Supplementary Information. Raw LC-MS data has been uploaded to MassIVE database (submission ID: MSV000098591).
